# Management of a Large Cerebral Abscess in Children Caused by Campylobacter gracilis: A Case Report and Review of the Literature

**DOI:** 10.7759/cureus.62744

**Published:** 2024-06-20

**Authors:** Yu Arakawa, Yusuke Yagi, Airi Mimoto, Yoshie Nishida, Shunsuke Kuwana, Eiichi Nakai, Tetsuya Ueba, Mikiya Fujieda, Yuka Yamagishi

**Affiliations:** 1 Department of Clinial Infectious Diseases, Kōchi Medical School Hospital, Kōchi University, Nankoku City, JPN; 2 Division of Infection and Prevention, Kōchi Medical School Hospital, Nankoku City, JPN; 3 Divison of Infection and Prevention, Kōchi Medical School Hospital, Nankoku City, JPN; 4 Department of Pharmacy, Kōchi Medical School Hospital, Nankoku City, JPN; 5 Department of Clinical laboratory, Kōchi Medical School Hospital, Nankoku City, JPN; 6 Department of Pediatrics, Kōchi Medical School, Kōchi University, Nankoku City, JPN; 7 Department of Neurosurgery, Kōchi Medical School, Kōchi University, Nankoku City, JPN; 8 Department of Clinical Infectious Diseases, Kōchi Medical School Hospital, Kōchi University, Nankoku City, JPN

**Keywords:** 16s rrna gene sequencing, carbapenem, oral flora, brain abscess, campylobacter gracilis

## Abstract

*Campylobacter gracilis* inhabits the gingival sulcus and has been reported to cause various periodontal diseases; it has rarely been reported to cause bacteremia. We describe a case of a two-year-old boy who presented with a consciousness disorder and was transferred to our hospital for treatment of a brain abscess. Magnetic resonance imaging (MRI) showed a 6-cm brain abscess in the right frontal lobe. Urgent drainage and antibiotic administration resulted in a favorable clinical course, and the patient was discharged on the 34th day of hospitalization. *Streptococcus anginosus* and *C. gracilis* were identified in the pus. Brain abscesses caused by *C. gracilis* have rarely been reported, which makes this a valuable case.

## Introduction

*Campylobacter gracilis*, formerly known as *Bacteroides gracilis*, is a non-motile, non-spore-forming, anaerobic, Gram-negative rod. This organism was reclassified into the genus Campylobacter in 1995 [1]. It predominantly resides in the gingival sulcus and is recognized for its role in the development of periodontal diseases. Although its role in oral infections is well documented, systemic infections attributed to *C. gracilis* are exceedingly rare, with few documented cases in the medical literature [2]. Dental infections account for approximately 10% of all brain abscesses, and the oral microbiome may act as a reservoir for intracranial infections [3]. Brain abscesses in children are medical emergencies that require prompt diagnosis and treatment. When caused by unusual pathogens such as *C. gracilis*, they present unique diagnostic and therapeutic challenges. Here, we report a rare case of a large brain abscess attributable to *C. gracilis* in a pediatric patient.

## Case presentation

A two-year-old boy experienced transient fevers of 37-38 °C, followed by vomiting, poor oral intake, and decreased activity approximately two weeks prior to hospital admission. One week before admission, the patient visited a hospital, where he received fluid therapy. However, the same symptoms persisted, and the patient presented with impaired consciousness when he visited another clinic on the day of admission. He was urgently transported to an emergency hospital, where computed tomography (CT) of the head revealed a suspected brain tumor. The patient was subsequently transferred to our hospital for further examination and treatment. He was born at term with no notable abnormalities or medical history.

**Figure 1:**
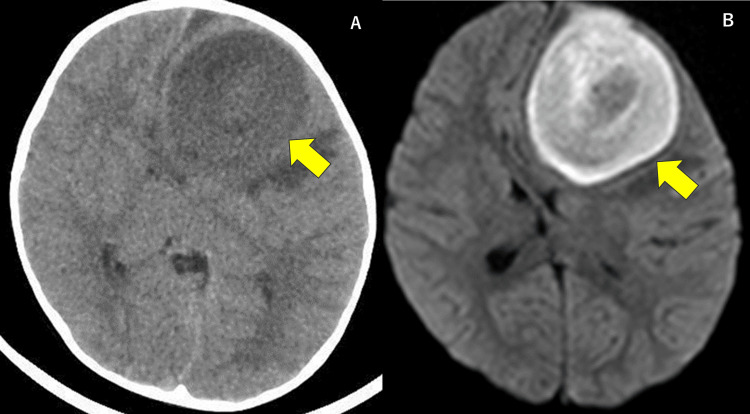
Imaging study of the brain on admission. (A) Head computed tomography (CT) without contrast; (B) diffusion-weighted magnetic resonance imaging (MRI). A 6 cm diameter mass lesion in the left frontal lobe with a midline shift was present (arrow).

**Figure 2:**
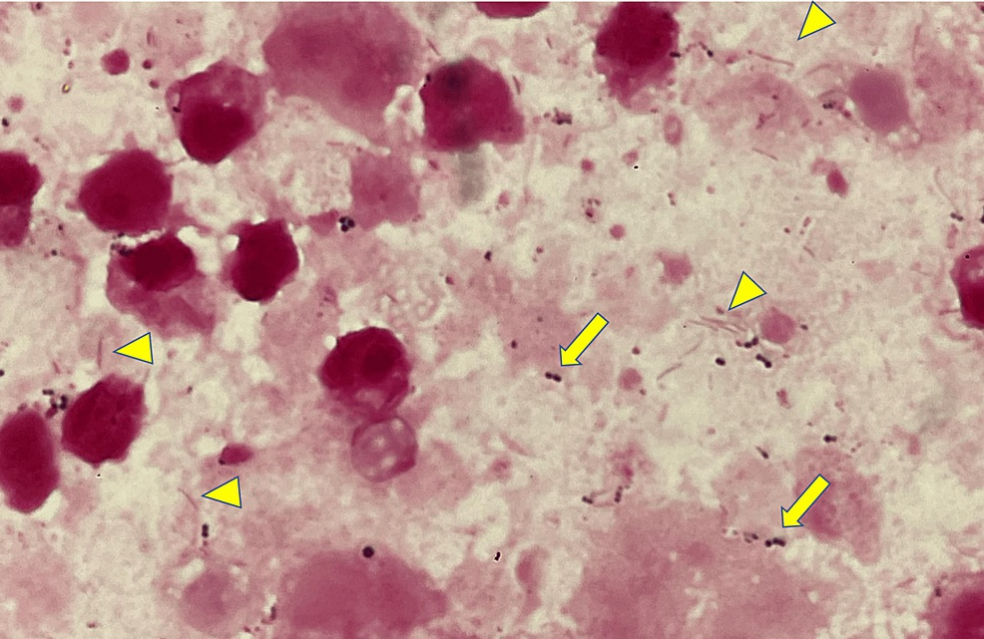
Gram staining of the pus from brain abscess in the present case. Gram-positive cocci (arrow) and Gram-negative rods (arrowhead) are shown.

On admission, his Glasgow Coma Scale (GCS) was 7 (eye-opening: 2, verbal response: 1, motor response: 4), body temperature was 36.1 °C, blood pressure was 176/97 mmHg, pulse rate was 70/minute, and blood oxygen saturation was 96% on ambient air. He appeared pale, and his pupils were 3/3 mm in size and reactive to light. There was bilateral hyperreflexia without significant left-right asymmetry. The results of the cardiovascular, respiratory, and abdominal examinations were normal. His blood test results revealed the following findings: white blood cell count, 20,900 cells/μL (with 83.0% neutrophils); platelet count, 571,000 cells/μL; and C-reactive protein level, 2.2 mg/dL. Serum creatinine, liver enzyme, immunoglobulin, and complement levels were normal. A brain CT revealed a low-density lesion measuring 6 cm in diameter in the left frontal lobe with a midline shift, and a hyperintense signal was observed in the same area on diffusion-weighted magnetic resonance imaging (MRI) (Figure 1A-1B). Therefore, a brain abscess was suspected, and an urgent craniotomy was performed for drainage of a brain abscess under general anesthesia. The abscess cavity was identified under ultrasound guidance, and a drainage tube was inserted. A total of 85 ml of pus was aspirated, which was grayish-white and had no characteristic odor. Gram-positive cocci and Gram-negative rods were detected in the pus (Figure 2). A combination therapy comprising meropenem and vancomycin was initiated for broad-spectrum coverage against Gram-positive streptococci, Gram-negative rods, and anaerobes.

Pus was cultured on blood agar medium, bromothymol blue (BTB) agar medium, chocolate medium (for aerobic culture), Gifu anaerobic agar medium (GAM; Nissui Pharmaceutical Co., Ltd., Japan), and BHK culture medium (for anaerobic culture). All media were incubated at 35 °C. On the second day of incubation, Gram-positive bacteria were found to have grown on the medium and were identified as *Streptococcus anginosus* by matrix-assisted laser desorption/ionization time-of-flight mass spectrometry (MALDI-TOF MS) (MALDI Biotyper ver. 9.0.0.0; Bruker Daltonics, Billerica, MA, USA). After 48 hour of culture, no growth other than *S. anginosus* was observed on the anaerobic medium. After 96 hours of culture, small colonies were observed on the BHK medium. It was difficult to identify the organism by using TOF-MS; therefore, species identification was performed using 16s rRNA gene sequencing (Appendix). Sequences were analyzed using the BLAST program (https://blast.ncbi.nlm.nih.gov/Blast.cgi; NCBI). The top three strains, all of *C. gracilis*, showed very high concordance with existing libraries (a 1029-bp fragment): 100.0% similarity with the KCOM3956 BLAST sequence strain (accession number MW725159.1), 99.90% similarity with the JCM10959 (LC383816.1) strain, and 99.90% similarity with JCM10958 (LC383815.1).

Table 1 displays the results of the drug susceptibility tests. Owing to poor growth, it was difficult to test drug susceptibility by the broth microdilution method, and the disk diffusion method and Etest® (BioMérieux, Inc., Marcy-l'Étoile, France) were used. The results were used as reference values owing to the absence of established interpretive criteria.

**Table 1:** Results of antimicrobial susceptibility testing. A: disk diffusion method, B: Etest®. PCG: penicillin G, ABPC: ampicillin, PIPC: piperacillin, CEZ: cefazolin, CPZ/SBT: cefoperazone/sulbactum, CMZ: cefmetazole, FMOX: flomoxef, IPM: imipenem, MEPM: meropenem, EM: erythromycin, CLDM: clindamycin, MINO: minocycline, LVFX: levofloxacin, MIC: minimum inhibitory concentration.

A: Disk diffusion method	
Antibiotics	Zone diameter (mm)
PCG	6
ABPC	12
PIPC	10
CEZ	30
CPZ/SBT	24
CMZ	6
FMOX	20
IPM	22
EM	20
CLDM	6
MINO	30
LVFX	6
B: Etest®	
Antibiotics	MIC (μg/mL)
MEPM	0.047
IPM	0.19

**Figure 3:**
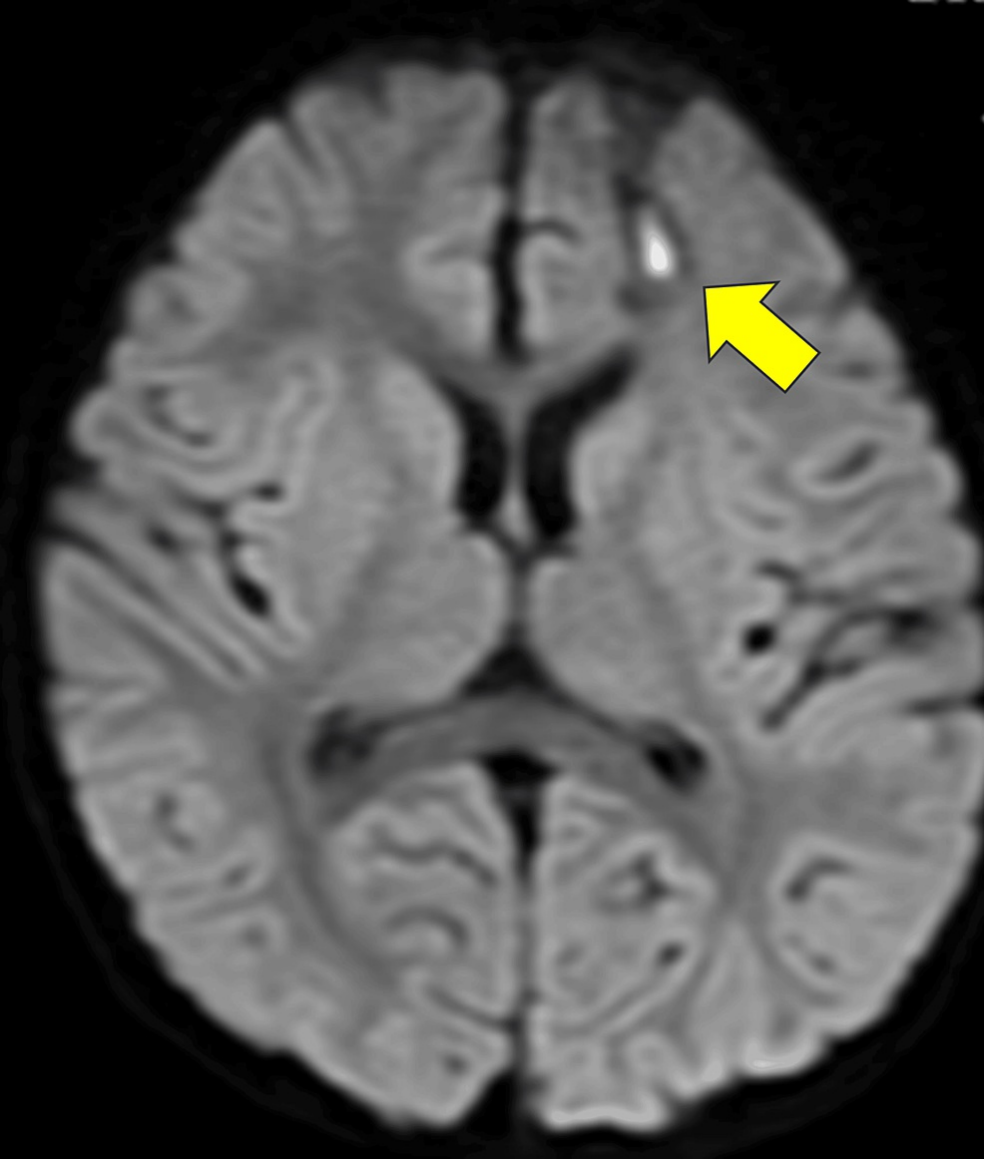
Diffusion-weighted magnetic resonance imaging of the brain on day 30 of admission. The abscess cavity has markedly reduced in size (arrow), and the midline shift has improved.

After surgery, the patient's consciousness level improved within a few days. Initially, the culture of the abscess revealed *S. anginosus*; however, the negative bacilli were poorly developed, and time was required for identification. On day 9 of hospitalization, a follow-up head MRI showed that the abscess had shrunk and changed to a cystic form. Vancomycin was discontinued when the Gram-negative rod was finally identified as *C. gracilis* and antimicrobial susceptibility results were confirmed. Echocardiography and dental and otolaryngological examinations revealed no obvious abnormalities. The patient's condition improved well, with a subsequent MRI on the 30th day after admission showing a reduction and only a slight high-intensity area remaining in the left frontal lobe (Figure 3). The total treatment duration was five weeks, after which the patient was discharged in a favorable condition.

## Discussion

*C. gracilis* is a non-motile, non-spore-forming, anaerobic Gram-negative rod, originally known as *B. gracilis*, which was reclassified into the genus Campylobacter in 1995 [1]. *C. gracilis* requires formate and fumarate for metabolism [2]. The genus Campylobacter is a candidate when a curved Gram-negative rod is isolated; however, this organism is not curved. Biochemical characterization tests indicated that common Campylobacter species are oxidase-positive, whereas *C. gracilis* is oxidase-negative [4].

*C. gracilis* is the most prevalent species of the Campylobacter genus in the oral cavity of healthy individuals. One study showed that *C. gracilis* is the most commonly found species among the Campylobacter species present in the oral cavity [5]. Furthermore, a study examining bacterial adherence to deciduous and permanent canines and molars in 40 children found that *C. gracilis* was present in 16.3% of permanent canines, 10.1% of permanent molars, 12.7% of deciduous canines, and 14.1% of deciduous molars [6]. Additionally, it has been reported that in shallow periodontal pockets, *C. gracilis* constitutes 37.5% of all Campylobacter species, which is the highest among them. In deep pockets, *C. gracilis* was the second most prevalent (27.1%) species, with the most prevalent species accounting for 43.4% [5]. These findings are supported by another study that identified *C. gracilis* as a causative agent for periodontal disease-related conditions [7]. These studies suggest that *C. gracilis*, which is commonly present in healthy oral cavities, can lead to systemic infections, including rare cases of brain abscesses.

Bacterial brain abscesses typically develop through one of three main mechanisms. First, continuous spread from neighboring sites of infection such as sinusitis, otitis media, mastoiditis, and dental infections. Second, hematogenous infections spread from distant infection sites, such as endocarditis, lung abscesses, and other pyogenic foci. Lastly, penetrating head trauma, neurosurgical procedures, or skull fractures can cause direct inoculation [3,8]. Of these, the routes of infection from the oral cavity to the central nervous system have been commonly identified as (1) direct spillover, (2) hematogenous spread, (3) via regional lymphatic vessels, and (4) indirect extraoral odontogenic transmission [9]. Hematogenous dissemination follows two principal pathways. Veins lacking valves, such as the facial, angular, and ocular veins, can serve as conduits for microorganisms to travel from the oral cavity to the cranium via the cavernous sinus. An alternative route involves systemic circulation. In this case, where the patient was immunocompetent and showed no evident infections in the oral cavity or elsewhere, we theorized that the infectious pathway to the central nervous system was venous in origin, suggesting that the pathogens leveraged venous circulation as a conduit for their dissemination.

*C. gracilis* causes infections at various sites; however, brain abscesses are rare. The most common infections identified included pleuropulmonary infections, intra-abdominal abscesses, and soft-tissue infections [10,11]. The infrequent reporting of brain abscesses, despite the common presence of this organism as an oral commensal, remains an intriguing paradox.

To date, there have been four documented cases of brain abscesses caused by *C. gracilis*. Two of these cases were reported in a retrospective survey in which *C. gracilis* was isolated as a contiguous source of infection from brain abscesses in patients with sinusitis. However, the details of these cases have been reported with much less specificity [10]. The remaining two cases are listed in Table 2. The first case (no. 3 in Table 2) was of a 35-year-old woman with postpartum conditions (two days prior to the symptomatic abscess) and unknown oral cavity status. This case was complicated by the co-isolation of multiple bacteria, which unfortunately resulted in the patient's death despite treatment. The second case (no. 4 in Table 2) was of a 60-year-old man with untreated dental caries and poor oral hygiene. This patient also had bacteremia due to *C. gracilis* and survived treatment [12].

**Table 2:** Summary of reported cases about brain abscess caused by C. gracilis. MEPM: meropenem, MMZ: metronidazole, VCM: vancomycin, *S. constellatus: Streptococcus constellatus.*

No.	Age	Gender	Underlying Conditions	Oral Cavity Condition	Blood Culture	Concurrently Detected Bacteria	Treatment	Prognosis	Reporting year	Reference
1	Unknown	Unknown	Sinusitis	Unknown	Unknown	Unknown	Unknown	Unknown	1985	1
2	Unknown	Unknown	Sinusitis	Unknown	Unknown	Unknown	Unknown	Unknown	1985	1
3	35	Female	Unknown	Unknown	Unknown	S. constellatus	MEPM +MMZ	Deceased	2008	3
4	60	Male	Untreated Dental Caries	Poor Hygiene	Positive	None	MEPM	Survived	2019	9
5	2	Male	None	Good	Negative	S. constellatus	MEPM+VCM	Survived	2024	Present case

The optimal antimicrobial therapy for *C. gracilis* infection has not yet been established. However, literature on antimicrobial susceptibility patterns shows conflicting results. In one study, the susceptibility of 23 *C. gracilis* isolates to penicillin was reported to be 67%, whereas the susceptibility to cephalosporins varied between 67% and 84% [10]. Another study indicated that all 10 examined strains were susceptible to a range of antibiotics, including amoxicillin/clavulanate, meropenem, cefoxitin, ceftriaxone, ceftizoxime, piperacillin, piperacillin/tazobactam, ticarcillin/clavulanate, metronidazole, and clindamycin [11]. In our case, drug susceptibility testing could only be performed using the disk diffusion method, and minimum inhibitory concentration (MIC) values could only be measured for carbapenems; therefore, treatment with meropenem, the susceptibility to which was considered reliable based on MIC ranges in previous reports [10], was continued.

Our case is the first reported instance of a pediatric brain abscess caused by *C. gracilis* and provides novel insights into the clinical spectrum of *C. gracilis* infections. Given the rarity of such infections, this report underscores the importance of considering *C. gracilis* in the differential diagnosis, particularly in pediatric cases with unusual presentations. The scarcity of documented *C. gracilis* infections highlights the significant gap in our understanding of their pathogenicity.

## Conclusions

In conclusion, we report a pediatric case of a large brain abscess attributable to *C. gracilis*. This case serves as a crucial call for further research and case reporting to enhance our knowledge and improve the management strategies for infection with this rare but potentially serious bacterium.

## Appendices

16S rRNA gene sequence method

The DNA extracted from the specimens was sequenced after library preparation. The bacterial colonies were suspended in nuclease-free water, boiled for five minutes, and used as templates. The primers used to amplify the bacterial 16S rRNA gene (approximately 1,500 bp) were 8UA (AGAGTTTGATCMTGGCTCAG) and 1485 B (TACGGTTACCTTGTTACGAC). Polymerase chain reaction (PCR) mixtures (25 μL) contained 10× PCR buffer, 2 mM deoxynucleotide triphosphates (dNTPs), 2.5 units/μL Taq DNA polymerase (Blend Taq™ -Plus-, TOYOBO, Shangai, China), 10 pmol of each primer, and 2 μL of template DNA. PCR included an initial denaturation step at 94 °C for four minutes, 35 cycles at 94 °C for 20 s, 58 °C for 30 s, 72 °C for one minute, and a final extension of 72 °C for five minutes. The amplified product was electrophoresed on a 1% agarose gel, extracted from the agarose gel, and purified using a Qiagen Gel Extraction Kit (QIAGEN, Germantown, MD, USA). The sequencing reactions were performed using the BigDye Terminator v3.1 Cycle Sequencing Kit, and the purified sequenced products were sequenced on an Applied Biosystems 3500 DNA analyzer (Applied Biosystems, Waltham, MA, USA).
